# Soft and flexible: core-shell ionic liquid resistive memory for electronic synapses

**DOI:** 10.1038/s41378-021-00305-7

**Published:** 2021-10-13

**Authors:** Muhammad Umair Khan, Qazi Muhammad Saqib, Mahesh Y. Chougale, Rayyan Ali Shaukat, Jungmin Kim, Jinho Bae

**Affiliations:** Department of Ocean System Engineering, Jeju National University, 102 Jejudaehakro, Jeju, 63243 Korea

**Keywords:** Electronic properties and materials, Electrical and electronic engineering

## Abstract

The human brain is the most efficient computational and intelligent system, and researchers are trying to mimic the human brain using solid-state materials. However, the use of solid-state materials has a limitation due to the movement of neurotransmitters. Hence, soft memory devices are receiving tremendous attention for smooth neurotransmission due to the ion concentration polarization mechanism. This paper proposes a core-shell soft ionic liquid (IL)-resistive memory device for electronic synapses using Cu/Ag@AgCl/Cu with multistate resistive behavior. The presence of the Ag@AgCl core shell in the liquid electrolyte significantly helps to control the movement of Cu^2+^ ions, which results in multistate resistive switching behavior. The core-shell IL soft memory device can open a gateway for electronic synapses.

## Introduction

Resistive switching memory (RSM) devices show promise for next-generation memory applications due to their simple fabrication process^1^, excellent stability^2,3^, and ultrafast switching performance^4^ at low operating voltages^5^. After the conceptualization of RSMs by Leon Chua in the 1970s^6^, Hewlett Packard Laboratories presented the foremost solid-state memory device^7^. Then, RSMs were also widely investigated to develop next-generation neuromorphic devices to mimic the brain^8–10^. Synapses are the most elegant memory and artifical intelligence network, in which different kinds of learning processes occur with the electrical activities of neurons^11,12^. Each neuron consists of a central cell body, which consists of narrow and long extensions (axons), each of which has indirect connections^13^. The cell membrane of each neuron is polarized with sodium (Na^+^) ions outside and potassium (K^+^) inside the cell, which communicates and results in the release of neurotransmitters during synapses^9,14^. The brain synapse process generates a signal of several tens of millivolts with high efficiency and low power^15,16^. Currently, researchers are trying to develop an artificial memory device that can operate as power efficiently as the human brain in which resistance change depends on the applied voltage. Liquid-based soft resistive memory can be applied to mimic the brain to perform synapses^9,17^.

Hence, researchers are applying various liquid materials, such as electrolytes, liquid metals, and ionic liquids (ILs), in RSMs due to their high flexibility, high conductivity, low cost, low viscosity, and easy device fabrication^18–21^. In addition, liquid-based RSMs work on the principle of transportation of cations and anions^17,19^. Soft RSMs show electrochemical metallization behavior and result in filamentary conduction, in which active electrodes such as Cu can inject Cu^2+^ metallic cations into the IL medium^19^. These metal cations (Cu^2+^) move to the cathode and are reduced by electrons. The precipitation of the active metals at the cathode leads to the growth of metal protrusion, which reaches the anode to form a highly conductive filament between the electrodes, and the conduction saturates due to ion polarization and results in resistive switching behavior^9^. These electrolytes will play an important role in the movement of cationic and anionic transportation to achieve neuromorphic behavior^9^.

**Fig. 1:**
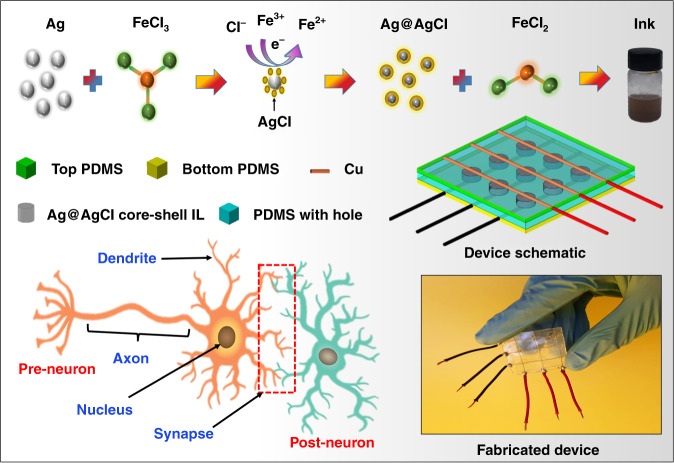
Material synthesis and device fabrication to compute the biological synapses. Ink preparation method of Ag@AgCl core-shell IL electrolyte and fabrication of soft and flexible memory cells for neuromorphic computing

This paper reports an Ag@AgCl core-shell (liquid electrolyte) synapse device with multistate resistive switching behavior, as shown in Fig. 1. Initially, iron(III) chloride (FeCl_3_) was used as an IL electrolyte to achieve resistive switching behavior. To control the conduction current to achieve multistate resistive switching behavior, Ag is introduced in a liquid electrolyte, which results in the formation of an Ag@AgCl core shell. The presence of a core shell significantly helps to control the device performance by limiting the movement of Cu^2+^ ions. The device presents a highly stable multistate resistive switching behavior at low operating voltage. We are confident that the proposed device is an excellent candidate for soft electronics to perform synapses similar to the human brain.

## Results and discussion

### Device structure and properties

Figure 1 shows the detailed process of the Ag@AgCl core-shell IL ink synthesis process, which is further utilized as an active material to perform biological synapses using memristive behavior. The presence of a core-shell IL controls the movement of Cu^2+^ ions, which results in multistate resistive switching behavior by applying a continuous sweep voltage and pulses with different frequencies, widths, and amplitudes. The liquid-based synapse memristive device is closer to the human neural model, which is based on the neurotransmitter, as shown in Fig. 1, in which two neurons are connected by a synapse^12^. The fabricated device is shown in Fig. 1 and can be used for soft and flexible electronics.

### Physical and electrical characteristics

**Fig. 2:**
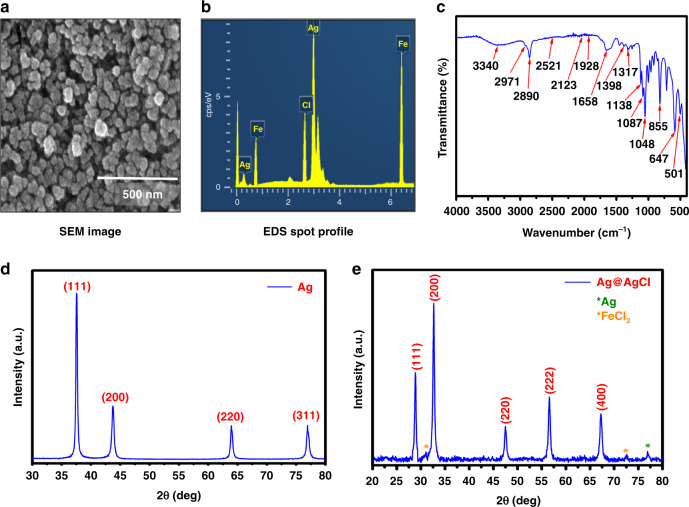
Material Characterisation of the Ag@AgCl coreshell ionic liquid for the fabrication of the **artificial** synaptic device. **a** SEM image, **b** EDS spot profile, and **c** FTIR analysis of the core shell. XRD pattern of **d** Ag and **e** Ag@AgCl

The preparation of the Ag@AgCl core shell can be observed using Field Emission Scanning Electron Microscope (FESEM) at a magnification scale of 500 nm, as shown in Fig. 2a. The average size of Ag@AgCl is ~100–120 nm. The confirmation of the core-shell structure can be observed from the energy-dispersive X-ray spectroscopy (EDS) spot profile, showing the presence of Ag, Fe, and Cl, as shown in Fig. 2b. Fourier-transform infrared spectroscopy (FTIR) analysis was performed to identify the presence of possible functional groups within the Ag@AgCl core shell^22^, as shown in Fig. 2c. The major infrared absorption peaks detected in the Ag@AgCl core shell were 3340, 2971, 2890, 1658, 1398, 1317, 1138, 1087, 1048, 855, and 647 cm^−1^. An intense band of alcohols and phenolic compounds with OH functional groups was observed at 3340 cm^−1^, whereas aliphatic and aromatic compound side-chain vibrations of C-H stretching were observed at 2971 and 2890 cm^−1^, respectively. The peak at 1658 cm^−1^ could be assigned to protein chains with stretching vibrations of C = O and -N-H- (amide I and amide II) bonds. Peaks located at 1317, 1138, and 1048 cm^−1^ might be attributed to the presence of stretching vibrations of amino groups, and carboxylic acids and alkyl halide stretching observed at 855 cm^−1^. The asymmetric deformation of CH_2_ and CH_3_ in proteins was observed at 1398 cm^−1^. The C-O-C bonds in polysaccharides were attributed to a peak at 1087 cm^−1^, which was typically found in the region between 1200 and 900 cm^−1^. The broad absorption band of the C-H bend alkyne was observed at 647 cm^−1^. Figure 2d shows the X-Ray Diffraction (XRD) patterns of silver (Ag) thin-film XRD, revealing polycrystalline behavior. The overwhelmingly concentrated peak positioned at 2*θ* = 38.02° relates to the face-centered cubic structure, revealing that the (111) planes of Ag were highly adapted and parallel to the substrate. Furthermore, we evaluated the average crystallite size by implementing Debye Scherrer’s equation, which was found to be 100 nm^23^. Figure 2e shows the XRD analysis of Ag@AgCl with peaks centered at 2*θ* = 28.5°, 32.6°, 45.7°, 55.4°, and 63.8° assigned to the (111), (200), (220), (222), and (400) planes, respectively. XRD shows some impurity peaks related to Ag and FeCl_2_ compounds with slightly lower intensities and the average crystallite was found to be 32 nm^24^.

### Electrical characteristics

**Fig. 3:**
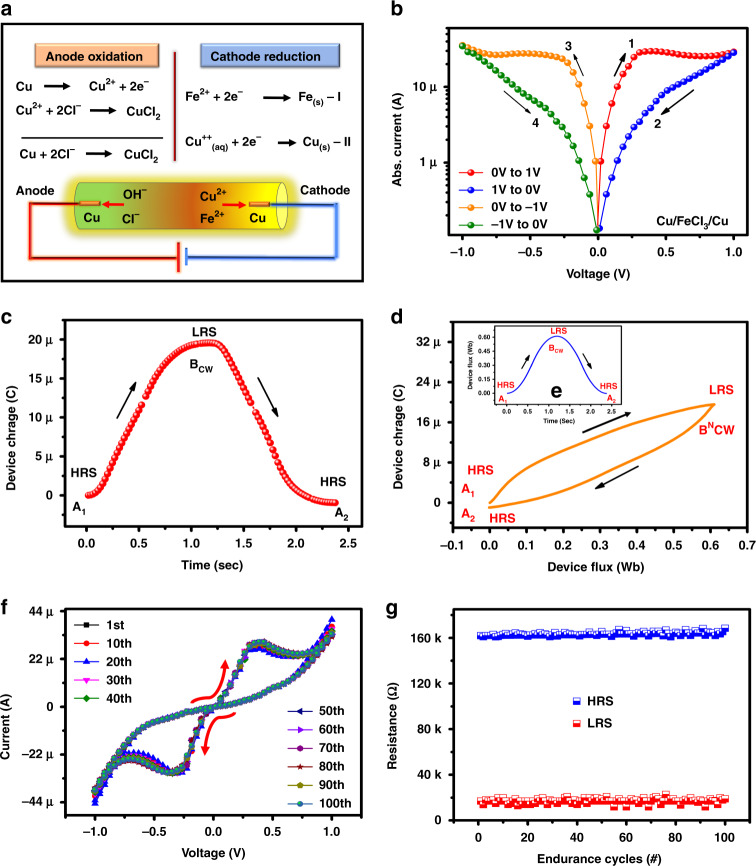
The working mechanism and electrical characterisation of the **resistive** memory device for soft and flexible electronics. **a** Ion conduction mechanism. **b** Semilog *I*–*V* curve of Cu/FeCl_3_/Cu. **c** Device dose–response with time, **d** charge–flux property of the device, and **e** device flux response with time. **f** 100 I-V sweep cycles and **g** device cycle-to-cycle endurance at a voltage read of 0.15

To achieve a higher OFF/ON resistance ratio, the FeCl_3_ ink concentration in glycerol was optimized, as discussed in Supplementary Fig. S1. The 0.2 wt % FeCl_3_ was added to glycerol, to prepare the electrolyte to investigate the resistive memory behavior. The main reason for the resistive switching in FeCl_3_-based ILs is due to ion movement between the anode and cathode (such as Fe^++^_,_ Cu^++^, and Cl^−^). Figure 3a shows the possible ionic transport mechanism of FeCl_3_. When a positive voltage was applied to the anode, oxidation was observed and the copper electrode changed to Cu^++^. The green color in the solution indicates that the Cu^++^ ions instead of Cu^+^ and 2Cl^−^ ions move to the anode, and copper II chloride (CuCl_2_) will be formed. On the cathode, there are two possibilities: (1) Fe^++^ ions move toward the copper electrode and (2) are reduced to Fe at the cathode. During the experiment, an orange color is observed in the electrolyte and this observation indicates that Fe is reduced at the cathode. Another possibility is the formation of Cu^++^ aqueous ions in the electrolyte, as discussed in our first observation, which may reduce to Cu at the cathode, as shown in Fig. 3a.

The FeCl_3_ IL was further characterized for resistive memory characteristics, showing resistive switching behavior, and the device passes from zero, as shown in Fig. 3b. To understand the memristive behavior, the device flux is shown in Fig. 3c and the device charge is discussed in Fig. 3e. The ideal memristor device shows nonlinearity in the charge and magnetic flux planes and passivity in the *I*–*V* plane, which means that the current should be zero at the origin. The ideal memristor *q*–*φ* characteristics should be nonlinear and single valued. To check whether the devices developed in this work are either memristors or memristives, we calculated the *q*–*φ* characteristics, as shown in Fig. 3d, with the help of experimental time-dependent *I*–*V* characteristics. The time-domain charge is given in Fig. 3c and flux characteristics are shown in Fig. 3e. The initial (A1), final-period (A2), turning (BCWN), and half-period (BCW) points represent the resistive switching states of the memristive device, as shown in Fig. 3c–e. The device was in the high resistance state (HRS) at points A1 and A2, and went into the low resistance state (LRS) at the BCWN point. The resistive memory device shows symmetric time-domain flux characteristics, as shown in Fig. 3e, because the voltage stimulus is symmetric in nature and its integration (*φ*(*t*)) must be an asymmetric function. However, the time-domain charge characteristics, as shown in Fig. 3c, of the FeCl_3_ resistive memory device were found to be asymmetric, which indicates that the pinched hysteresis loop of the developed device was asymmetric. Similarly, the nonlinear *q*–*φ* characteristic suggests that nonideal memristor properties are observed in the FeCl_3_ resistive memory device, as shown in Fig. 3d. Cycle-to-cycle repeatability was performed for more than 100 cycles, as shown in Fig. 3f, and the device showed stable repeatability. The *I*–*V* endurance is shown in Fig. 3g with an HRS of 161.9 kΩ and an LRS of 15.4 kΩ at a voltage read of 0.15 V with OFF/ON resistance ratio of ~10.51. To achieve multistate resistive switching, Ag nanoparticles are added to FeCl_3_, which results in the formation of an Ag@AgCl core shell. The formation of a core shell helps to achieve multistate resistive switching to perform neuromorphic computing. The detailed ink preparation method for the neuromorphic device is given in the “Materials and Methods” section.

**Fig. 4:**
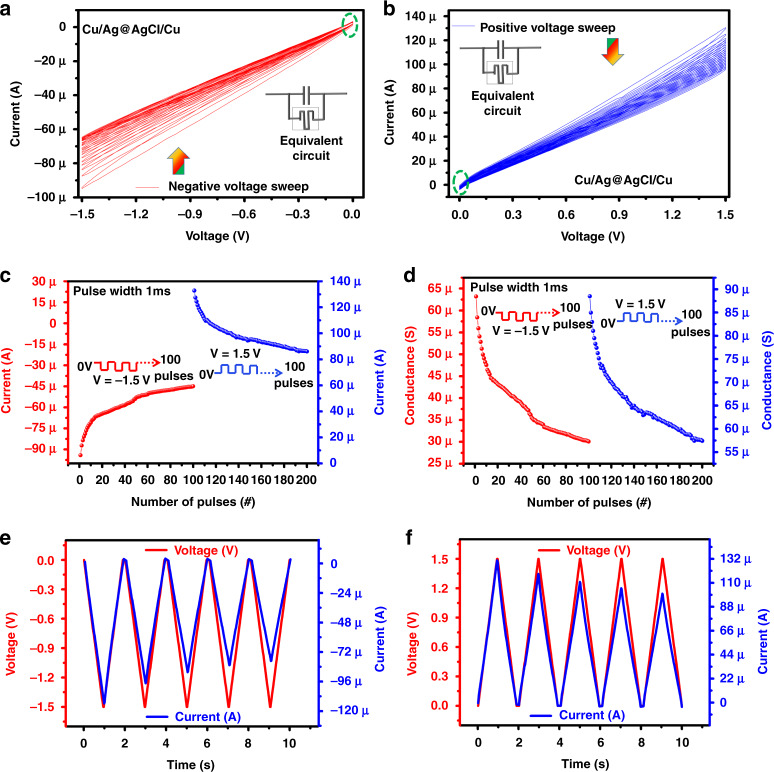
The multistate **resistive switching** behaviour showing **conductance** change and triangular pulse response to observe current change. **a** Multistate *I*–*V* curve of Cu/Ag@AgCl/Cu in the **a** negative voltage region and **b** positive voltage region. The **c** current and **d** conductance response of the device for a pulse width of 1 ms with a reading pulse amplitude of −1.5 V on negative and 1.5 V on positive voltage region. The triangular pulse response in **e** negative and **f** positive voltage regions

The human brain is composed of interconnected neurons, where the neurons communicate with each other by synaptic plasticity^25^. Synaptic plasticity between presynaptic and postsynaptic neurons is responsible for the efficient functioning of the human brain^3^. As depicted in Fig. 1, the transmission process of neurotransmitters (Na^+^ or Ca^2+^ ions) initiates neuronal spikes that control synaptic plasticity between presynaptic neurons and postsynaptic neurons^13,26^. Similarly, the memristor device can also mimic biological synaptic plasticity by gradual variation in the resistance state, using repeated pulse sequences to modulate conduction in the switching layer^27^. To achieve synaptic behavior, an Ag@AgCl core shell was used in the IL. Our core-shell soft resistive memory device synapse is equivalent to the parallel combination of a capacitor with a memristor, as shown in Fig. 4a, b. The capacitance^7,28^ behavior can be observed near the origin of the positive voltage region, as shown in Fig. 4a, and the negative voltage region, as shown in Fig. 4b. Notably, the presence of the Ag@AgCl core shell and Cu^2+^ ions has a remarkable influence on the device performance under a dual voltage sweep. Multistate resistive switching was achieved by applying dual voltage sweeps on the positive and negative voltage regions, as shown in Fig. 4a, b. In the positive and negative voltage regions, the current decreased with an increase in the number of voltage sweeps. Therefore, the resistance state of the Cu/Ag@AgCl/Cu device gradually increases with consecutive voltage sweeps and the conductance of the device decreases. Thus, the electrical characteristics of the core-shell soft resistive memory device are related to the typical electrical properties of memristive devices used as synapses.

The multistate resistive switching is due mainly to the presence of Ag@AgCl core-shell ions in the IL, which resists the flow of Cu^++^ ions, and second, ion metallization at the cathode and anode. The multistate characteristics of the core shell-based IL device were observed during a continuous voltage sweep of 0 V → +1.5 V → 0 V. Then, the core-shell IL device shows turn-OFF behavior when the voltage range is 0 V. During a voltage sweep from 0 V → +1.5 V, Cu^++^ ions enter the ionic solution and the diffusion flux helps to increase the flow of ions and moves toward the cathode. At the same time, a very small amount of Cu^++^ ions react with 2Cl^−^ ions at the anode and result in the formation of CuCl_2_ (oxidation) and release of 2*e*^−^. However, the movement of Cu^++^ can be reduced by the Ag@AgCl core shell, which was the main reason for the multistate resistive switching. During voltage sweep from +1.5 V → 0 V, the Cu^++^ ions move toward the cathode and accept 2*e*^−^, which results in a reduction of Cu at the cathode. In this whole process, ionic flux saturates, because ion transportation proceeds with the diffusion of concentration gradient flux, and a decrease in current appears beyond a critical point. The movement of Cu increases at a certain voltage and then the movement of ions decreases, due to which breakage of conduction filaments, and diffusive flux and concentration gradient flux increase until ions are depleted on the Cu electrode, resulting in the creation of a higher resistance value (metallization). Similarly, this sweep cycle repeats every time and results in multistate resistive switching.

To further emulate a biological system, a more sophisticated pulse scheme was implemented to mimic the spike rate-dependent plasticity (SRDP), in which the current and conductance were obtained by applying 100 repetitive pulses of different voltages with a pulse width of 1 ms to the Cu/Ag@AgCl/Cu device, as shown in Fig. 4c, d. The gradual variation in the current and conductance with pulses is similar to a variable synaptic weight in biosynapses. In the Ag@AgCl core-shell device, a similar phenomenon also exists after inverting the polarity on the positive or negative voltage region, as shown in Fig. 4c, d. These results indicate that the Ag@AgCl core-shell soft IL and flexible synaptic device behave like brain neuronal activation. Thus, the electrical characteristics are related to the typical electrical properties of memristives used as synapses. Figure 4e, f show the measured current under a five triangular voltage sweep with a period of 2 s. The conducting current varied with time, as shown in Fig. 4e, f. We can see that the device current nonlinearly decreases in the negative and positive voltage sweep regions.

**Fig. 5:**
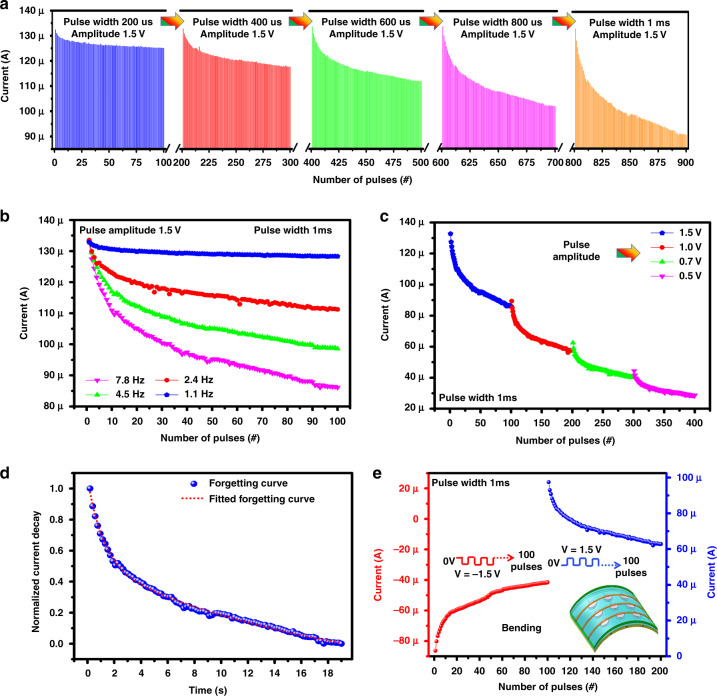
The synaptic behaviours based on pulse width, **frequency** and voltage and device bending effect. **a** Plasticity characteristics of the artificial synapse with different pulse widths of 200 μs, 400 μs, 600 μs, 800 μs, and 1 ms. **b** Frequency response at 1.1, 2.4, 4.5, and 7.8 Hz. **c** The different pulse amplitude responses at 1.5, 1.0, 0.7, and 0.5 V. **d** The forgetting curve of the neuromorphic device. **e** Bending test of the device under a continuous pulse train

The synaptic weight of the Cu/Ag@AgCl/Cu core-shell IL artificial synapse device is defined by the current of the multistate switching and can be modulated by successive stimuli of externally applied pulses of different widths. Therefore, pulse widths of 200 μs, 400 μs, 600 μs, 800 μs, and 1 ms were investigated by applying 100 consecutive synaptic pulses with an amplitude of 1.5 V, as shown in Fig. 5a. In the case of a pulse width of 200 μs with 100 consecutive pulses, no obvious decrease in the current was observed. However, by increasing the pulse width to 1 ms, the synaptic current changes significantly from 136 to 97 μA compared to a pulse width of 200, 400, 600, and 800 μs. This phenomenon is basically due to better ionic diffusion at higher pulse widths, which provides a higher change in current.

The SRDP of the soft core-shell resistive memory device was examined by varying the frequency, as shown in Fig. 5b, and different amplitudes of the pulses, as shown in Fig. 5c. For 100 consecutive pulses with an amplitude of 1.5 V and a width of 1 ms, there is almost no increase in the response current at a given frequency of 1.1 Hz. However, when the frequency increases to 7.8 Hz, the current decreasing rate (135–95 µA) is larger than the current decreasing rate of previous conditions with lower frequencies (1.1, 2.4, and 4.5 Hz), as illustrated in Fig. 5b. This behavior is related to the competition between the interval between two pulses and the relaxation time of the artificial synapse, which can be considered metallization of the electrode, and its rate increases with higher frequencies. When the amplitude of 100 pulses on voltage is 1.5, 1, 0.7, and 0.5 V with a pulse width of 1 ms, the current decrease rate becomes even smaller in the lower voltage range, as illustrated in Fig. 5c, which corresponds to slow movement of diffusion flow of ions and results in a decrease in the rate of metallization.

The decay of the synaptic weight of the Cu/Ag@AgCl/Cu core-shell IL artificial synapse device is shown in Fig. 5d. To obtain the forgetting curve, presynaptic input pulses were applied with *V* = 1.5 V and pulse width = 1 ms by keeping the device in the LRS state. As a result, the postsynaptic current is monitored with time. The forgetting curve of the device nearly fits with the forgetting curve of the human brain, as given in Eq. (1), where *W*(*t*) is the synaptic weight of the device at time *t*, *W*_e_ is the synaptic weight in the stable state, *K* is a constant, and *τ* is the relaxation time constant^13^.

1 $$W\left( t \right) = W_{\mathrm{e}} + K\exp \left( { - t/\tau } \right)$$

This experimental part is based on the demonstration of device bending, which can open a gateway for future IL device structures to intergrade in soft electronics. The bending and flexible nature of the device was due to the usage of the main component of the Ag@AgCl core-shell electrolyte and polydimethylsiloxane (PDMS) substrate, as shown in Fig. 5e. The bending state current was obtained by applying 100 repetitive pulses of 1.5 V and −1.5 V in the positive and negative voltage regions, respectively, with a pulse width of 1 ms, as shown in Fig. 5e. The flexible nature of the device helps to achieve stable bending performance and can be used as a synaptic device. This study opens a gateway for soft and liquid-switching media for electronic synapses.

**Fig. 6:**
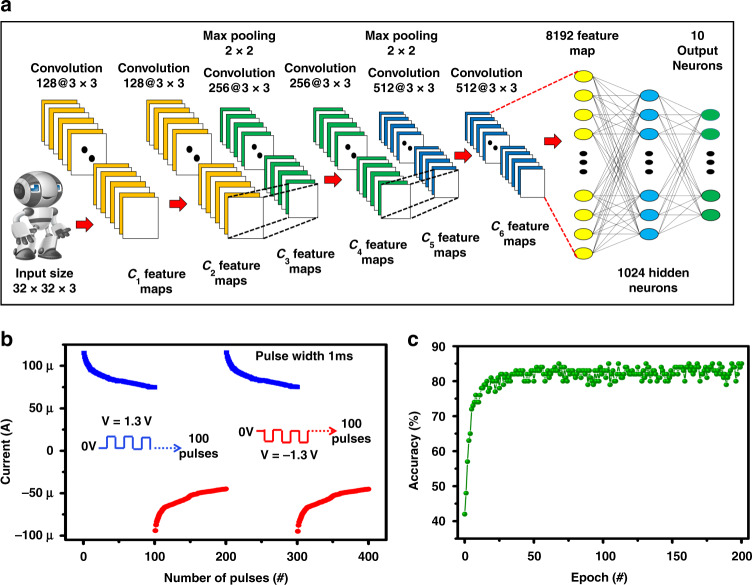
The implementation of convolutional neural network using the synaptic behaviour of memristive device to recognise CIFAR-10 data set. **a** The convolutional neural network. **b** Synaptic plasticity under a continuous pulse train. **c** Simulated accuracy of each epoch during training

Figure 6a shows the neuromorphic simulation structure. The input image data based on CIFAR-10 are used for training and testing. The simulation is composed of six convolutional neural networks and two fully connected layers^29^, as shown in Fig. 6a. The 100 continuous input pulses of amplitude +1.3 V and −1.3 V, respectively, with a pulse width of 1 ms were applied to investigate the current change in the positive and negative regions, as shown in Fig. 6b. The data given in Fig. 6b were applied to perform deep learning, to investigate the system accuracy using DNN_NeuroSim_V2.1^29^. The analog weight nonlinearities are ~3.74 for a positive pulse current and 4.17 for a negative pulse current for the data given in Fig. 6b, and the cycle-to-cycle variation is measured as 0.003. As the simulation result shows in Fig. 6c, the maximum network learning accuracy converges to 85% under 200 epochs.

**Fig. 7:**
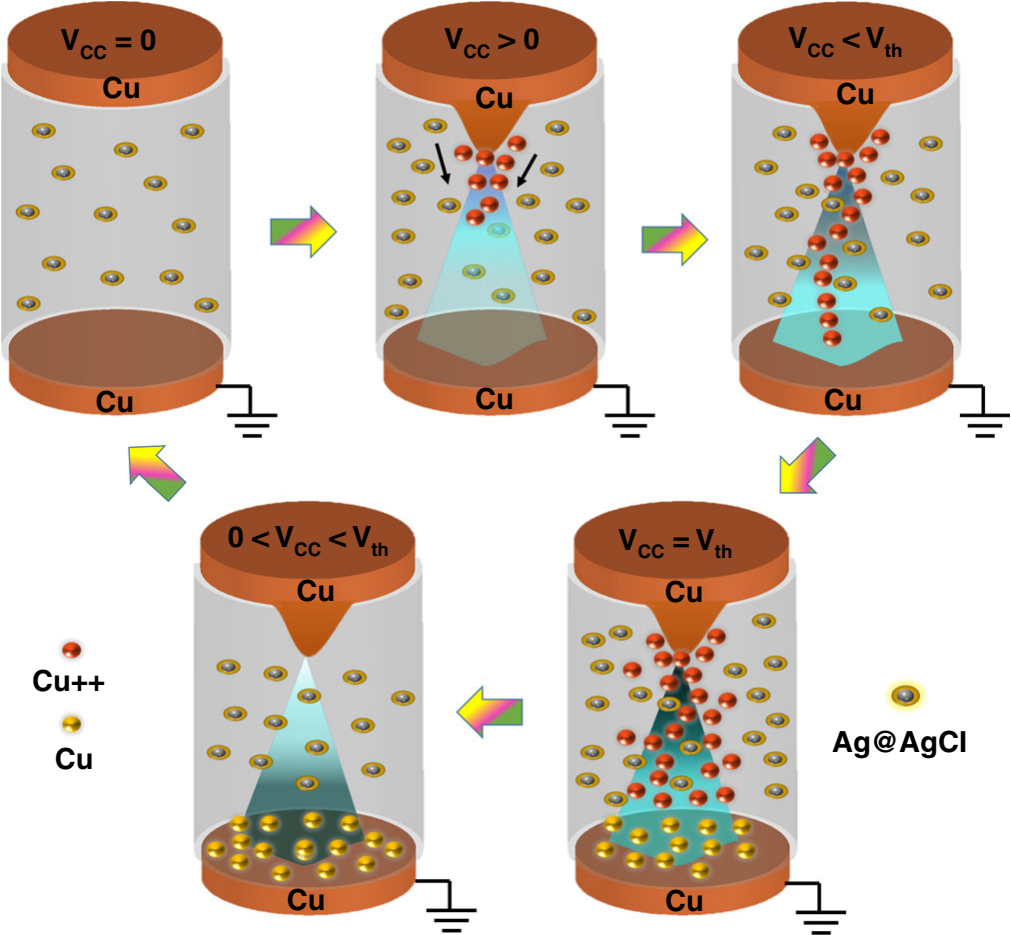
Proposed model for working mechnism of the artifical synaptic device using Ag@AgCl corshell ionic liquid. The working mechanism of the core-shell soft ionic liquid neuromorphic device

This mechanism part, as shown in Fig. 7, illustrates only the flow of Cu ions in a working mechanism of core shell-based IL. The multistate resistive switching is due to the movement of Cu ions, which can be regulated by tuning the amplitude of the applied voltage. The multistate resistive switching characteristics of the core shell-based device are illustrated in Fig. 7, in which a positive voltage sweep (V_cc_) was applied on the top electrode to the anode Cu electrode and the ground was applied to the bottom cathode Cu electrode. During forward voltage sweep 0 V → 1.5 V, initially at *V*_cc_ = 0, there is no movement of Cu ions and the device remains in the HRS state. If *V*_cc_ > 0 V, then the core-shell IL device shows turn-OFF behavior. The IL device with “ON” and “OFF” states depends solely on the time during which conduction filaments form between the anode and cathode. During the *V*_cc_ > 0 V state, Cu^++^ ions start entering an ionic solution and the diffusion flux helps to increase the flow of ions. When the voltage range is between *V*_cc_ < threshold voltage (*V*_th_), ions move toward the cathode and, at the same time, a very small amount of Cu^++^ ions react with OH^−^ ions at the anode, resulting in the formation of Cu(OH)_2_ (oxidation) and releasing 2*e*^−^. At a voltage state of *V*_cc_ = *V*_th_, the Cu^++^ ions move toward the cathode and accept 2*e*^−^, which results in a reduction of Cu at the cathode, as illustrated in Fig. 7. At the same time, the presence of Ag@Agcl core-shell nanoparticles restricted the movement of Cu^++^ ions, which became the main reason for multistate resistive switching. In this whole process, ionic flux saturates, because ion transportation proceeds with the diffusion of concentration gradient flux and a decrease in current appears beyond a critical point. During reverse voltage sweep 1.5 V → 0 V, movement of Cu ions increases at a certain value of voltage and then the movement of ions decreases, due to which breakage of the conduction filament occurs and the off state at voltage state 0 V < *V*_cc_ < *V*_th_ was due to diffusive flux and concentration gradient flux increases until ions are depleted on the Cu electrode resulting in the creation of a higher resistance value at *V*_cc_ = 0 V. This process will repeat during every voltage sweep, which results in the multistate resistive switching behavior.

## Conclusions

In conclusion, this study has proposed a core-shell soft IL-resistive memory device for electronic synapses with a Cu/Ag@AgCl/Cu structure. The mechanism of the core shell-based device is highly dependent on ion concentration polarization, which is the main reason for synaptic behavior. Synaptic plastic behavior was observed in both positive and negative voltage regions to investigate the pulse width, frequency, and pulse amplitude. The mechanical bending test demonstrates stable synaptic behavior. We are sure that core-shell IL devices can open a gateway for the development of a highly efficient and soft brain-mimicking system.

## Materials and methods

### Synthesis of Ag@AgCl core-shell IL

FeCl_3_ hexahydrate crystallized, glycerol solution, and Ag nanoparticles (50 wt%) in tetrahydrofuran (THF) solvent were purchased from Sigma Aldrich for the preparation of Ag@AgCl core shells. Ink preparation is presented in Fig. 1. We prepared a core shell by dropwise adding 5 wt % FeCl_3_ solution to glycerol and 50 wt % Ag nanoparticle solution to THF solutions at a ratio of 1 : 1. After this reaction, the mixture was kept at room temperature until its color changed from black to brown^30^. Color changes are observed in the reaction, which confirms the Ag@AgCl core shell with iron II chloride (FeCl_2_), as shown in Fig. 1.

### Fabrication of the memristor

PDMS was purchased from Dow Corning for the preparation of the mold. The fabrication process is explained in Fig. 1, in which a PDMS mold is prepared by mixing curing agent and PDMS in a 1 : 10 ratio and cured at 80 °C for 4 h. In the top and bottom PDMS molds, Cu wires with a thickness of 1 mm were used for electrodes at both ends (anode and cathode). A porous mold with a hole size of 3 mm was used to hold the core-shell IL with a thickness of 200 µm. Detailed fabrication of the PDMS mold is given in our previously reported work^19,20^. In the final step, Ag@AgCl core-shell IL was used as a resistive switching medium and was filled in PDMS mold using a syringe and two electrodes as anode and cathode used as contacts, and PDMS helps to avoid liquid leakage in a channel between electrodes.

### Device characterizations

Current-voltage (I-V) and neuromorphic characterization of the device was performed with the KEYSIGHT B2902A source measuring unit. The surface morphology of Ag@AgCl core-shell nanoparticles was analyzed through TESCAN MIRA3 STEM and the core-shell element compositions were analyzed using EDS. The chemical and structural information of the Ag@AgCl core shell was investigated by FTIR using a Bruker IFS 66V spectrometer. The crystal structure of the FeCl_3_, Ag, and Ag@AgCl core shell was characterized by XRD diffraction with a tube target of Cu.

## Supplementary information

Supplementry Information
