# Supplementary material for: Soft and flexible: core-shell ionic liquid resistive memory for electronic synapses
Source: Microsyst Nanoeng. 2021 Oct 13;7:78. doi: 10.1038/s41378-021-00305-7 (PMC8514441; doi:10.1038/s41378-021-00305-7)
Supplement: Supplementary file 1 — Supplementry Information [file 41378_2021_305_MOESM1_ESM.docx]

**Supplementary information:**

**Soft and flexible: core-shell ionic liquid resistive memory for electronic synapses**

Muhammad Umair Khan, Qazi Muhammad Saqib, Mahesh Y. Chougale, Rayyan Ali Shaukat, Jungmin Kim, and Jinho Bae*

Department of Ocean System Engineering, Jeju National University, 102 Jejudaehakro, Jeju 63243, Korea

^*^E-mail: [baejh@jejunu.ac.kr](mailto:baejh@jejunu.ac.kr)


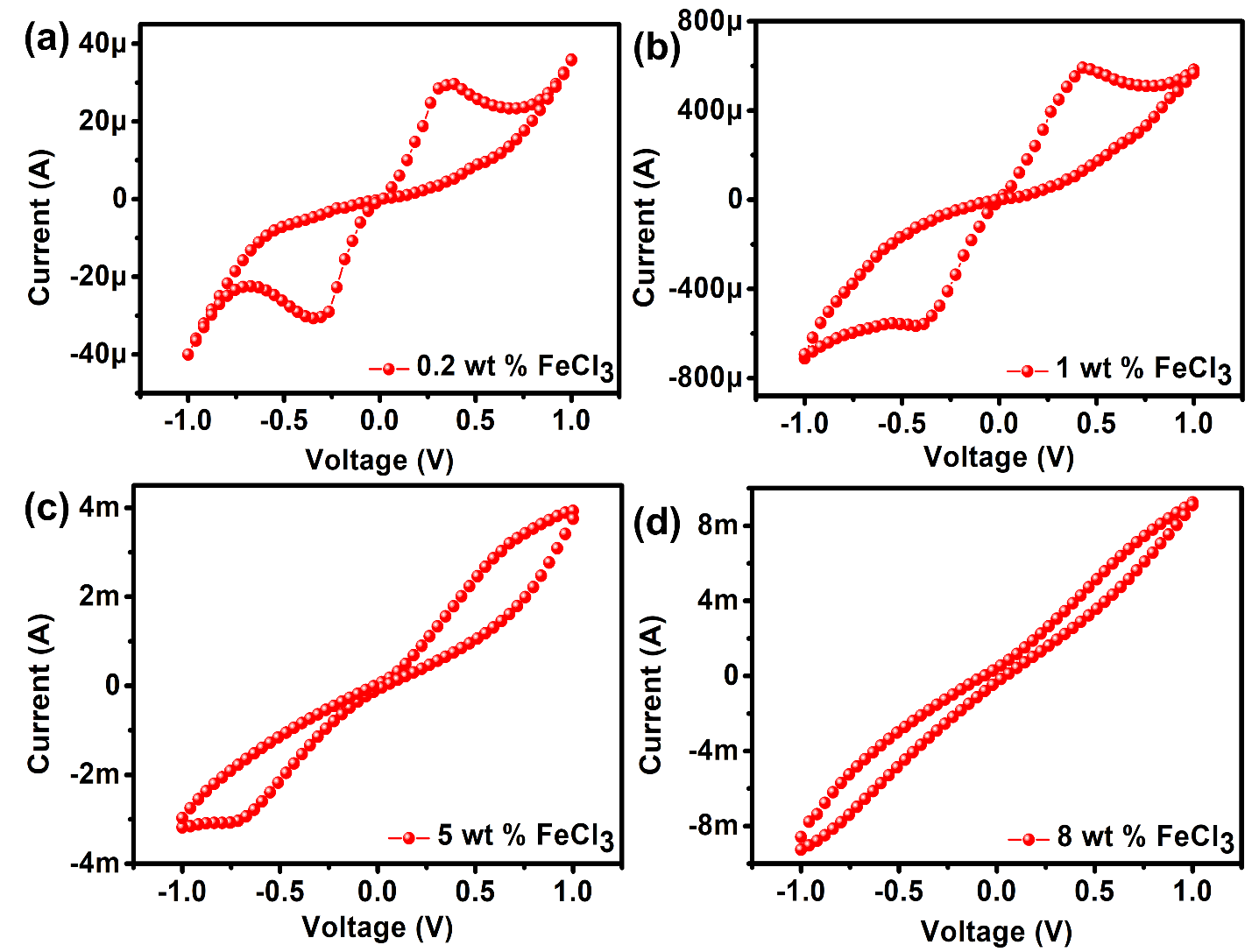


**Fig. S1:** The IV behavior using: (a) 0.2 wt % FeCl_3_, (b) 1 wt % FeCl_3_, (c) 5 wt % FeCl_3_, and (d) 8 wt % FeCl_3_ in glycerol.
